# Microglia and Synapse: Interactions in Health and Neurodegeneration

**DOI:** 10.1155/2013/425845

**Published:** 2013-12-11

**Authors:** Zuzana Šišková, Marie-Ève Tremblay

**Affiliations:** ^1^German Center for Neurodegenerative Diseases (DZNE), Ludwig-Erhard-Allee 2, 53175 Bonn, Germany; ^2^Axe Neurosciences, Centre de Recherche du CHU de Québec, Département de Médecine Moléculaire, Université Laval, 2705 Boulevard Laurier, Québec, QC, Canada G1V 4G2

## Abstract

A series of discoveries spanning for the last few years has challenged our view of microglial function, the main form of immune defense in the brain. The surveillance of neuronal circuits executed by each microglial cell overseeing its territory occurs in the form of regular, dynamic interactions. Microglial contacts with individual neuronal compartments, such as dendritic spines and axonal terminals, ensure that redundant or dysfunctional elements are recognized and eliminated from the brain. Microglia take on a new shape that is large and amoeboid when a threat to brain integrity is detected. In this defensive form, they migrate to the endangered sites, where they help to minimize the extent of the brain insult. However, in neurodegenerative diseases that are associated with misfolding and aggregation of synaptic proteins, these vital defensive functions appear to be compromised. Many microglial functions, such as phagocytosis, might be overwhelmed during exposure to the abnormal levels of misfolded proteins in their proximity. This might prevent them from attending to their normal duties, such as the stripping of degenerating synaptic terminals, before neuronal function is irreparably impaired. In these conditions microglia become chronically activated and appear to take on new, destructive roles by direct or indirect inflammatory attack.

## 1. Physiological Conditions

Microglial cells derive from primitive myeloid progenitors (which originate from the yolk sac) invading the central nervous system (CNS) during embryonic development. As a consequence, they are the only immune cells that permanently reside in the CNS [1–3]. In the healthy CNS, microglia occupy minimally overlapping territories in which they continuously survey their environment by structurally remodeling their ramified processes on a time scale of minutes [4–8]. These surveillant microglia can respond rapidly to any pathological stimulus resulting from injury or disease by transforming their morphology and functional behavior [9–12]. Traditionally, these changes in the microglial phenotype are referred to as *microglial activation*. Activated microglia have the capacity to proliferate, migrate, and release reactive oxygen species, neurotoxins, and proinflammatory and anti-inflammatory cytokines. These activated microglia can secrete trophic factors, present antigens to T cells, and phagocytose pathogens, degenerating cells, and inflammatory debris [9–11, 13–15]. In addition, they can separate presynaptic terminals from the postsynaptic neuronal parts in a process known as *synaptic stripping *[16, 17]. It has long been thought that most of these vital functions can only be performed by activated microglia.

However, in recent years, several fundamental insights into the roles of microglia have been provided with new, noninvasive approaches that have allowed the study of their function while avoiding their activation [18–21]. Surprisingly, surveillant microglia were found to (i) eliminate neuronal precursors in the cortical proliferative zones and to (ii) regulate the density of dendritic spines in the hypothalamus, with consequences on the masculinization of adult copulatory behavior; (iii) the functional maturation of glutamatergic receptors in the hippocampus; and (iv) the activity of tectal neurons in the zebrafish [22–25]. In the mature CNS, surveillant microglia were also found to (v) phagocytose newborn cells during adult hippocampal neurogenesis and (vi) regulate glutamatergic synaptic transmission in the hippocampus [26, 27].

**Figure 1:**
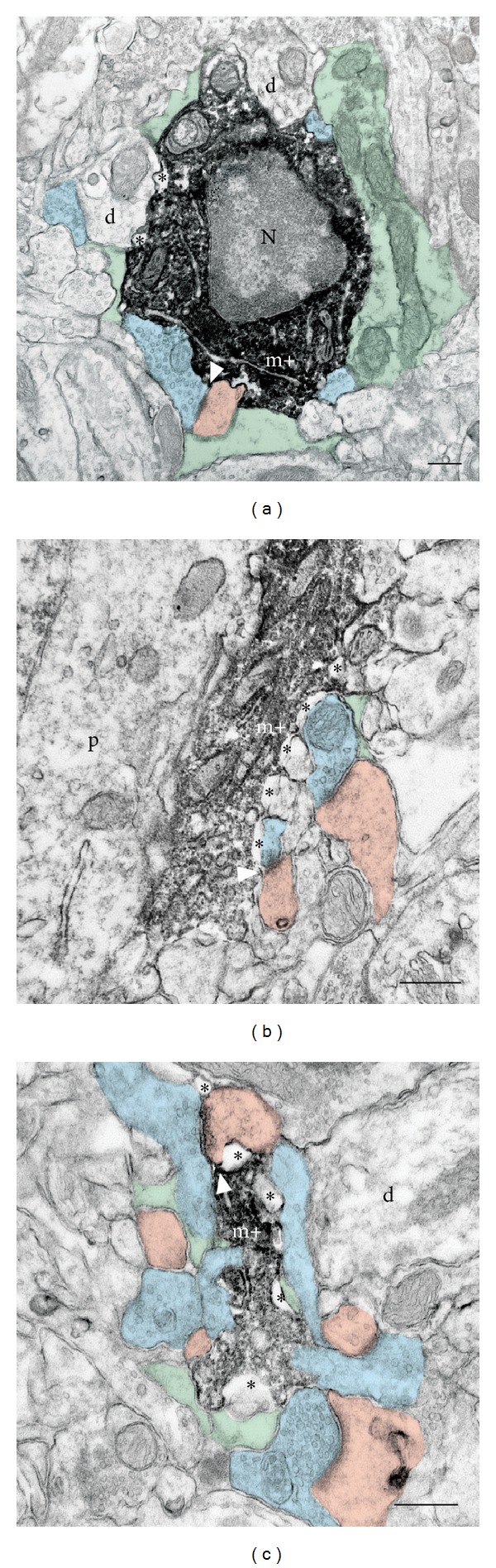
Ultrastructural relationships between microglia and synaptic elements in physiological conditions. Electron microscopy examples of Iba1-immunostained microglial (m+) cell body (a), as well as large (b) and small (c) processes, showing direct juxtaposition with axonal terminals (in blue), dendritic spines (in pink), astrocytic processes (in green), and synaptic cleft (arrow) in adolescent mouse visual cortex. d: dendrite; N: nucleus; p: perikaryon; asterisks: extracellular space. Scale bars: 250 nm. Reproduced from Tremblay et al. [30].

**Figure 2:**
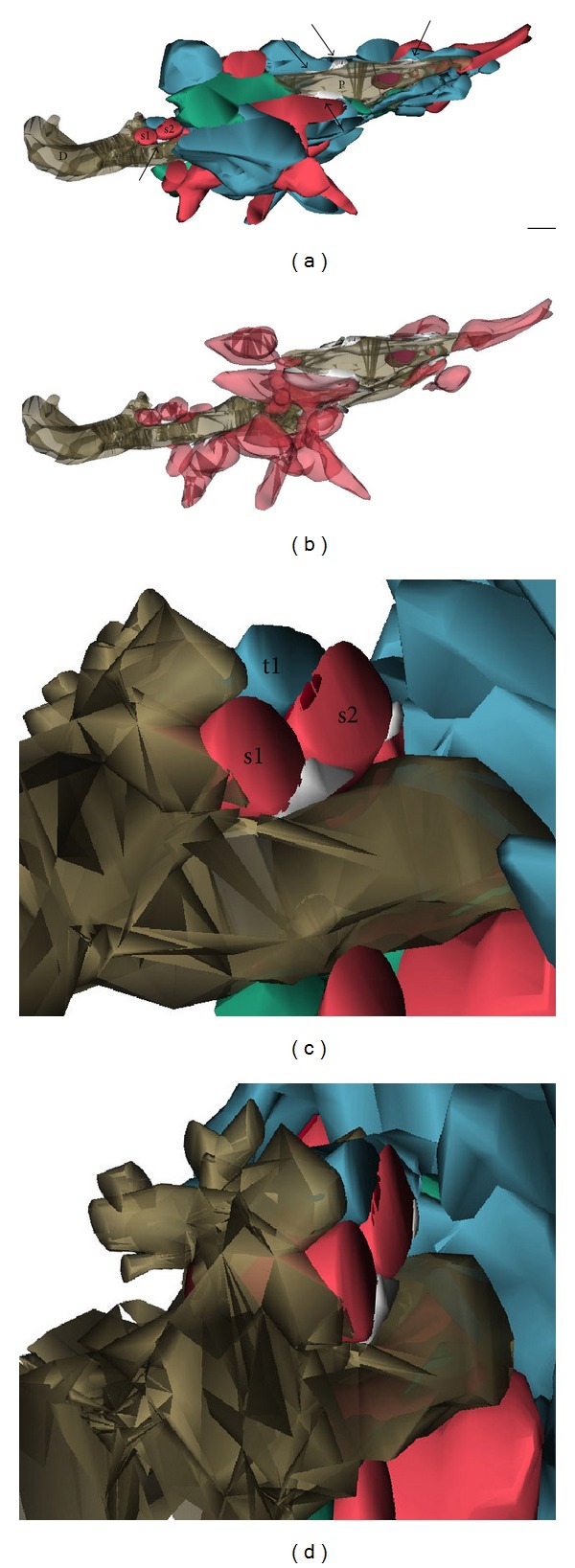
Three-dimensional reconstruction of microglial relationships with multiple synaptic elements at multiple synapses in physiological conditions. (a) Partial reconstruction of a microglial proximal process (P), cut in transverse, and a distal protrusion (D), cut longitudinally, in adolescent mouse visual cortex. The purple element indicates a phagocytic inclusion. The process and protrusion simultaneously contact multiple axonal terminals (in blue), dendritic spines (in red), and perisynaptic astrocytic processes (in green) and are distinctively surrounded by extracellular space pockets (arrows) of various sizes and shapes (in white). (b) Additional view showing only microglia, dendritic spines, and extracellular space. (c) and (d) Insets illustrating the three-dimensional relationships between the distal protrusion, one axonal terminal (t1), two dendritic spines (s1 and s2; postsynaptic density in dark red), and a pocket of extracellular space (in white), which are partially reconstructed. For clarity, an astrocytic process was removed from the scene. Scale bar: 250 nm. Reproduced from Tremblay et al. [30].

With relation to synapses, it was also recently revealed that surveillant microglia directly contact synaptic elements and eliminate particular subsets of axonal terminals and dendritic spines, depending on changes in neuronal activity and sensory experience, both in the developing and mature brain [21, 28–32]. Microglial interactions with synaptic elements are prevalent [29–32], with almost all of the microglial processes (~94%) juxtaposing axonal terminals, dendritic spines, perisynaptic astrocytic processes, or synaptic clefts and ~68% of all microglial processes contacting more than one synaptic element simultaneously [30] (Figure 1). Morphological specializations resembling finger-like protrusions wrapping around dendritic spines were described based on electron microscopy with three-dimensional reconstruction (Figure 2). Clathrin-coated pits are also frequently encountered among microglial processes, synaptic structures, and perisynaptic astrocytic processes, suggesting direct exchanges of molecular signals between microglia and synapses by clathrin-mediated endocytosis of membrane-bound receptors and ligands [30]. Importantly, when microglial phagocytosis is compromised during early postnatal development, a sustained impairment of synaptic connectivity is present until adulthood [28]. These results imply that, in addition to the immune defense of the brain, microglia-specific activity plays a crucial role in the refinement of neuronal circuits.

Microglial involvement in pruning of synapses, that is, an activity-dependent process required for the maturation of neuronal circuits, is now well-established during postnatal development. Importantly, in the past years, synaptic pruning was found to be determined by the microglial chemokine receptor CX_3_CR1 and the classical complement cascade, including, most notably, signaling between the microglial complement receptor 3 (CR3) and the neuronal opsonin C3 [21, 28]. A similar role was recently proposed in the mature CNS, in the experience-dependent remodeling of neuronal circuits, but the molecular cues remain largely unknown, besides ATP signaling through purinoceptors [4, 6, 18]. In the mature brain, phagocytic inclusions showing ultrastructural features of axonal terminals and dendritic spines were frequently observed inside microglial cell bodies or processes, in both the visual and auditory cortices [31]. The engulfed synaptic elements displayed various signs of health, such as an electron-lucent cytoplasm, intact organelles, and cytoskeletal elements, in contrast to the apoptotic elements that are phagocytosed during adult neurogenesis or in contexts of disease [26, 33–36]. Importantly, microglia-synapse interactions were also found to be regulated by neuronal activity, with the phagocytic inclusions becoming more prevalent during manipulations of visual experience [30, 31], including a period of light deprivation followed or not by reexposure to light, a paradigm associated with increased neuronal circuit remodeling and dendritic spine elimination [37–39]. Other mechanisms by which microglia could eliminate synaptic elements may notably include the release of proteases, such as cathepsins, matrix metalloproteinases (MMPs), and tissue-plasminogen activation, as these cells were found to be uniquely surrounded by pockets of extracellular spaces of various sizes and shapes, suggesting their ability to remodel the geometry of the extracellular space locally and thus the concentration of signaling molecules in the synaptic environment [30]. These proteases have been associated *in vitro *with dendritic spine growth and increases in synaptic strength and *in vivo *with dendritic spine motility and elimination, as well as experience-dependent plasticity [18].

**Figure 3:**
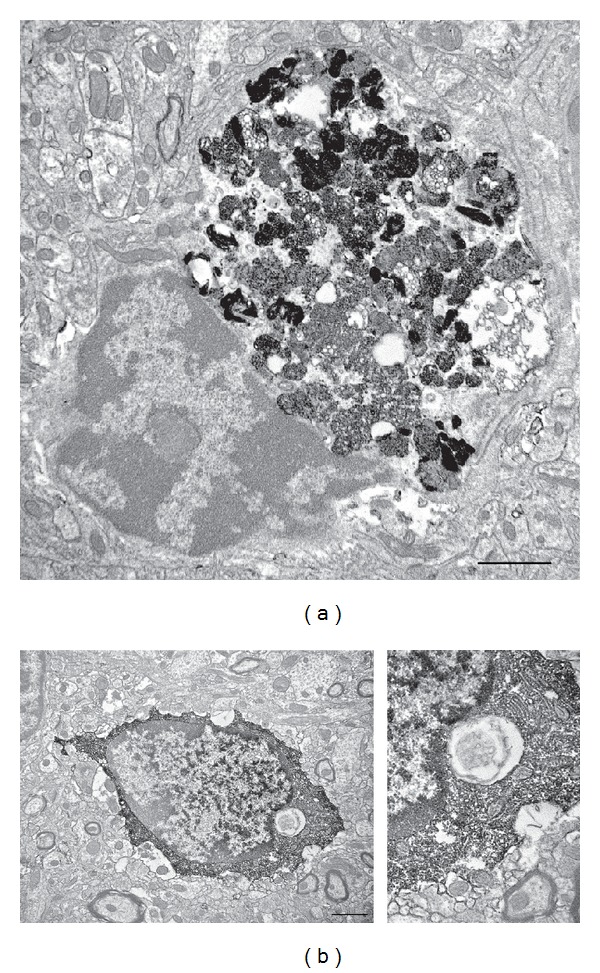
Microglial accumulation of phagocytic inclusions during aging and age-associated loss of sensory function. Examples of Iba1-immunostained microglia containing (a) accumulation of cellular inclusions from the phagocytic elimination of neurons or glial cells and (b) a single cellular inclusion that resembles an axonal terminal with clearly visible synaptic vesicles (inset) in the visual cortex of a 20-month-old mouse with age-associated loss of visual function. Scale bars: 1000 nm. Reproduced from Tremblay et al. [31].

During normal aging, the microglial population may be more heterogeneous, displaying variable morphology and different distribution within the brain parenchyma [31]. It has been revealed that ~20% of all microglia in the visual and auditory cortex are completely filled with cellular debris (including axonal terminals, dendritic spines, lysosomal vacuoles, and lipopigments) akin to fat granule cells or gitter cells (see Figure 3). Another distinct feature of the aging brain is the prevalence of microglial interactions with degenerating neurons and synapses, which are identified by their electron-dense, dark ultrastructural contents. The prevalence of phagocytic inclusions and microglial contacts with degenerating elements may be particularly exacerbated by the loss of visual or auditory function [31]. Furthermore, numerous microglial processes protruding into the synaptic cleft have been observed, suggestive of synaptic stripping [17, 31]. In addition, enlargement and thickening of the microglial cell body, increased granulation, impairment of remodeling, and retraction of microglial processes have been described in various brain regions [12, 31, 40–42]. These compromised microglial functions might cause impaired reaction to neuronal abnormalities, in addition to impairing synaptic plasticity, thereby exacerbating the cognitive decline associated with aging [43, 44].

## 2. Neurodegenerative Diseases

Age is the largest risk factor for the development and progression of neurodegenerative diseases. Several of them, including Alzheimer's and prion diseases, share a common element of pathology: the misfolding and aggregation of otherwise soluble proteins, which are normally mostly enriched at synapses [45]. Over the past two decades, there has been some progress in our understanding of these complex pathologies with respect to the mechanisms underlying neuronal dysfunction. While neuronal death is the final, irreversible outcome in such diseases, the loss of synapses has emerged as a major correlate of cognitive decline [46]. Within the scope of this review, the following section will examine the interactions of microglia with synapses in Alzheimer's (AD) and prion diseases.

### 2.1. Alzheimer's Disease

In AD, deposition of misfolded extracellular and intracellular proteins is correlated with neuronal dysfunction and loss leading to clinical symptoms of dementia. The principal structural unit of the extracellular deposits is a relatively small peptide, amyloid *β*-protein (A*β*), which is capable of forming long, insoluble amyloid fibrils that accumulate in deposits known as *senile plaques *during the evolution of the disease [46]. A number of studies have implicated the oligomeric A*β* forms alone as capable of impairing synaptic function, even in the absence of amyloid fibrils or plaques [47, 48]. Furthermore, it appears that the synapses are the initial targets and their loss is the major correlate of cognitive impairment [49–51].

Following amyloid plaque formation, activated microglia accumulate in its vicinity [52]. Although their exact function remained elusive for some time, recent studies have implied a dual role for microglia in AD pathology. *In vivo *imaging showed that A*β* plaques can form surprisingly quickly (over 24 hours) and microglia might help to restrict their growth [53]. In agreement with this finding, microglial depletion has been linked to increased plaque load in the brain, indicating that microglia might be neuroprotective by removing A*β* [54]. Plaque removal is accomplished by secretion of proteolytic enzymes and via receptors, such as class A scavenger receptors, the receptor for advanced glycation end products (RAGE), and *β*1 integrins [55–58]. It has been suggested that Ccr2, a chemokine receptor expressed on microglia, might also facilitate the removal of A*β* in the early stages of AD [59]. Following immunization therapy, significant amounts of A*β* within the microglial cells of AD patients were observed during postmortem analysis [60]. Several other studies have also documented that A*β* clearance might be a crucial recovery-promoting mechanism [61–63]; however, in most cases, the precise identity of the phagocytic cells involved remains yet to be determined.

Interestingly, observations contradictory to the aforementioned findings were made by another study following ablation of microglia for up to 4 weeks. Despite a dramatic reduction in microglial numbers, no change in the amount or morphology of A*β* deposits was observed and neuronal damage appeared unaltered [64]. It is worth considering that longer periods of microglial depletion and earlier onset might be required to better understand their role in plaque removal [65]. Nevertheless, this might be challenging to accomplish, because a rapid, efficient repopulation resembling that of the endogenous microglial population occurs in the brain following chemical depletion of microglia [66].

**Figure 4:**
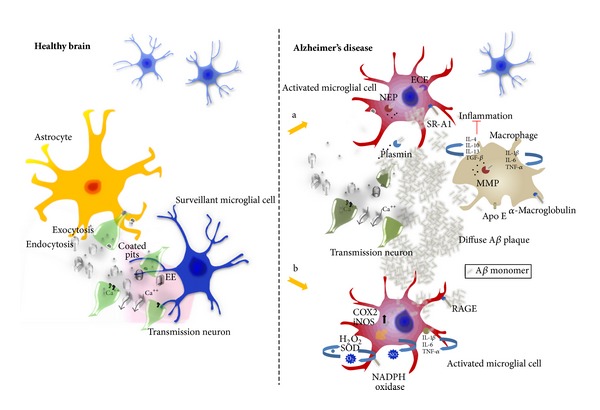
The role of microglial cells in Alzheimer's disease. Increased accumulation of A*β* peptides is thought to trigger a variety of pathological events, which subsequently compromise neuronal function. For example, A*β* molecules are known to interact with neurotransmitter receptors, disrupt synaptic and mitochondrial function, and promote neuronal proapoptotic signalling. Injured neurons release a variety of factors that together with A*β* accumulation trigger microglial activation. (a) Extracellular A*β* is taken up and degraded by microglia via receptors such as *β*-integrins, various enzymes including MMPs, and other uptake-mediating molecules, for example, ECE and NEP. Additionally, macrophages can degrade A*β* molecules and, together with microglia, help restrict amyloid plaque formation in the brain. These activities might represent an important recovery-promoting mechanism. (b) In response to the deposition of A*β* and the release of chemoattractants from injured neurons, activated microglia can release a range of proinflammatory mediators such as cytokines (IL-1*β*, IL-6, and TNF-*α*) and induce generation of reactive oxygen species. Although this initial response might be an attempt to protect the brain, these proinflammatory activities are believed to be a major detrimental factor, worsening the disease outcome.

On the other hand, microglia are activated by A*β* to produce cytokines, chemokines, and neurotoxins and may therefore exacerbate neuronal degeneration [57, 67, 68]. One of the examples is the chemokine receptor CX_3_CR1 for fractalkine/CX_3_CL1, a ligand expressed in neurons that is known for recruiting CX_3_CR1-expressing microglia to injured neurons [69]. In a mouse model of AD, microglial CX_3_CR1 knockout prevented neuronal loss, indicating that microglia might be involved in neuronal elimination during neurodegeneration [70]. In addition to extracellular protein deposition, intracellular neurofibrillary tangle formation is another major component of AD pathology associated with processes of microglial activation. In an animal model of tauopathy mimicking neurofibrillary tangle formation, microglial activation coincided with the elimination of synapses; however, no evidence of synaptic stripping was provided [71]. Although some studies have shown that microglia can ingest apoptotic neurons and neuritic blebs [72], the precise nature of the involvement of microglia in the events underlying synaptic degeneration and elimination remains to be determined. Taken together, microglia appear to be either beneficial by removing A*β* or detrimental through their proinflammatory activities, thereby likely worsening the disease outcome (Figure 4).

### 2.2. Prion Diseases

**Figure 5:**
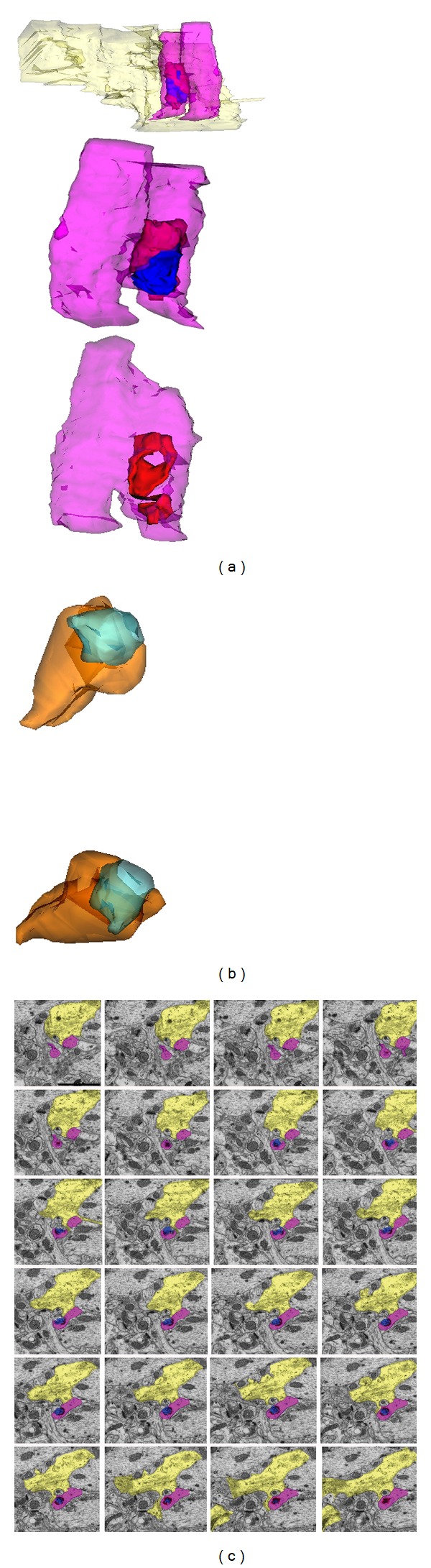
Three-dimensional reconstructions of degenerating synapses in the stratum radiatum of hippocampus in prion disease. (a) Rotations of a synaptic profile generated from 46 consecutive sections; the material originating from the presynaptic terminal (in blue) remains outside and inside (engulfed by the PSD (in red)) of the dendritic spine (in purple). Note the presence of one astrocytic process (in yellow) in proximity but not engaged with the degenerating terminal. The first 24 consecutive sections, from which the profile was generated, are illustrated in (c). (b) Rotations of a synaptic profile from 20 consecutive sections. The presynaptic element (in blue) appears internalized by the dendritic spine head (in orange); however, a fine strand of material originating from the presynaptic element remains in association with the extracellular space and is not within the encircling PSD of the spine. (c) Electron micrographs of serial sections illustrating a degenerating synaptic terminal in the stratum radiatum neuropil. The cytoplasm of the presynaptic element (in blue) is electron-dense in all sections; although the synaptic vesicles are still visible, the presynaptic element is disconnected from the projecting axonal terminal and remains arrested and almost completely engulfed by the PSD (in red) of the dendritic spine (in purple). A process of one astrocytic cell (in yellow) is in close proximity. Scale bar: 1000 nm. Reproduced from Šišková et al. [89].

Prion diseases are a group of progressive neurodegenerative conditions affecting both humans and animals. The hallmark pathological features, which are associated with accumulation of a misfolded isoform of the cellular prion protein (PrP^Sc^), are spongiform degeneration of the brain with extensive neuronal loss, dendritic and synaptic abnormalities, and astrogliosis [73–77]. The misfolded, protease-resistant protein was long implicated in the demise of neurons; however, recent evidence suggests that it might be the protein oligomers, as in AD, that precipitate the synaptic dysfunction [78]. In animal models, the appearance of behavioral abnormalities is associated with a loss of synapses in the hippocampus [79, 80] before the loss of neurons occurs [80, 81]. The presynaptic terminal has been postulated as the initiation site of synaptic demise, followed by dendritic spine degeneration [75, 79, 81, 82]. Similar to AD, microglia undergo a functional transformation associated with a typical change of their morphology [83–85]. However, their molecular fingerprint appears to be anti-inflammatory [86], akin to a macrophage involved in a phagocytic process [87]. At the present time, it is not known whether microglia become activated because of the PrP^Sc^ accumulation or because of the synaptic changes [88], nor it is known what leads to the anti-inflammatory phenotype they appear to adopt. Taken together, it seems likely that microglia with this functional profile might be involved in synaptic stripping. While the aforementioned remains elusive in AD, a series of recent discoveries have pointed to its absence in prion disease [89, 90]. So far, no stripping of either pre- or postsynaptic elements has been observed by electron microscopy; however, engulfment and digestion of neuronal corpses have not been excluded. The density of glutamatergic synapses in the hippocampus gradually decreases, while the remaining synapses hypertrophied, similar to AD [91–93]. A striking feature of the synaptic pathology is the progressive change in the curvature of the PSD, which, as the disease evolves, gradually envelopes the presynaptic element, which appears to be internalized by the dendritic spine. Surprisingly, the loss of synapses is not associated with microglia or astrocytes; instead, it appears to be a neuron-autonomous event (Figure 5 [89, 94]). The subsequent loss of spines has been associated with the development of varicosities on dendritic shafts. Interestingly, only the persistent spines are lost, while the transient spines appear to be unaffected [95].

However, it appears that synaptic degeneration is not a ubiquitous early event in prion disease and that the synaptic vulnerability to toxic protein depends prominently on the structure and function of the target neurons. Brain region-specific presynaptic and postsynaptic degenerative processes independent of microglia were described by a recent study; while synaptic pathology was present in the hippocampus, virtually no synapses had been lost in the cerebellum [90].

Taken together, while activated microglia are thought to exacerbate chronic neurodegenerative conditions such as prion diseases [96], their involvement in synaptic loss via synaptic stripping appears to be unlikely given the recent findings. However, the decision between synaptic elimination and its maintenance in the degenerating brain might be regulated by a more subtle, indirect mechanism, such as extracellular signaling, the nature of which remains to be clarified.

## 3. Conclusion and Perspectives

Together, these recent discoveries demonstrate that microglia preserve the health of neuronal circuitry by continuous surveillance and dynamic adaptation to changes in neuronal activity and sensory experience. Upon activation, microglia may intervene by eliminating particular subsets of synaptic structures (e.g., axonal terminals and dendritic spines) if a threat to neuronal integrity arises.

In neurodegenerative conditions associated with pathological accumulation of misfolded proteins, such as AD and prion diseases, chronic activation of microglia might exacerbate ongoing degenerative processes. In AD, the activation of microglia appears to have contradictory consequences. On the one hand, microglial activation appears to be beneficial by facilitating the removal of neuronal corpses, inflammatory debris, and A*β* plaques. However, the secretion of neurotoxins and proinflammatory activities associated with microglial activation may worsen the disease outcome. In prion diseases, microglial activation occurs relatively early during the disease process and it is one of the few correlates of synaptic and behavioral abnormalities. While chronic activation of microglia likely worsens the disease outcome, microglia were not observed to engage in synaptic removal via synaptic stripping.

Taken together, all essential cognitive functions, such as learning, memory, and language, rely on the experience-dependent remodeling of neuronal circuits, a process in which microglial interactions with synapses play a role of paramount importance. It is a pressing matter to understand the exact nature of these interactions, because in neurodegenerative conditions it is at the synapse where the fate of a neuron seems to be decided.
